# Intelligent Clinical Decision Support System for Managing COPD Patients

**DOI:** 10.3390/jpm13091359

**Published:** 2023-09-06

**Authors:** José Pereira, Nuno Antunes, Joana Rosa, João C. Ferreira, Sandra Mogo, Manuel Pereira

**Affiliations:** 1 INOV Inesc Inovação—Instituto de Novas Tecnologias, 1000-029 Lisbon, Portugal; jose.pereira@inov.pt (J.P.); nuno.f.antunes@inov.pt (N.A.); joana.rosa@inov.pt (J.R.); 2 Instituto Universitário de Lisboa (ISCTE-IUL), ISTAR (Information Sciences, Technologies and Architecture Research Center), 1649-026 Lisboa, Portugal; 3 Logistics, Molde University College, NO-6410 Molde, Norway; 4 Departamento de Física, Universidade da Beira Interior, 6201-001 Covilhã, Portugal; sipmogo@gmail.com; 5 Hope Care, S.A, 2510-216 Óbidos, Portugal; manuel.pereira@hope-care.pt

**Keywords:** chronic obstructive pulmonary disease (COPD), decision support system (DSS), intelligent clinical decision support system (CIDSS), health remote monitoring system (HRMS), triage validation module (TVM)

## Abstract

Chronic obstructive pulmonary disease (COPD) is the third leading cause of death worldwide. Health remote monitoring systems (HRMSs) play a crucial role in managing COPD patients by identifying anomalies in their biometric signs and alerting healthcare professionals. By analyzing the relationships between biometric signs and environmental factors, it is possible to develop artificial intelligence models that are capable of inferring patients’ future health deterioration risks. In this research work, we review recent works in this area and develop an intelligent clinical decision support system (CIDSS) that is capable of providing early information concerning patient health evolution and risk analysis in order to support the treatment of COPD patients. The present work’s CIDSS is composed of two main modules: the vital signs prediction module and the early warning score calculation module, which generate the patient health information and deterioration risks, respectively. Additionally, the CIDSS generates alerts whenever a biometric sign measurement falls outside the allowed range for a patient or in case a basal value changes significantly. Finally, the system was implemented and assessed in a real case and validated in clinical terms through an evaluation survey answered by healthcare professionals involved in the project. In conclusion, the CIDSS proves to be a useful and valuable tool for medical and healthcare professionals, enabling proactive intervention and facilitating adjustments to the medical treatment of patients.

## 1. Introduction

### 1.1. COPD Introduction and Definition

According to the World Health Organization (WHO), chronic obstructive pulmonary disease is one of the most deadly major lung diseases and the third leading cause of death worldwide [1]; the organization further indicates that COPD was responsible for about 3.24 million deaths in 2019. The Portuguese Society of Pulmonology [2] estimates that 5.42% of individuals in Portugal between the ages of 35 and 69 suffer from COPD. According to the Portuguese Lung Foundation [3], COPD was responsible for approximately 2834 fatalities in the country. The same organization calculates that in 2019, this illness cost the economy EUR 1.6 billion.

COPD is caused by airway obstruction. The most common symptoms of COPD are coughing, wheezing, and dyspnea (shortness of breath). Patients often seek medical attention only when the disease reaches an advanced stage, as it is a condition that progresses slowly.

Initially, the disease presents as a cough accompanied by increased sputum production. However, as it progresses, it can lead to repeated episodes of acute bronchitis and respiratory infections. As the disease develops further, shortness of breath becomes more frequent, even with seemingly minor tasks, such as talking and performing daily hygiene. Shortness of breath is most noticeable during activities that require physical effort.

### 1.2. Importance of COPD Management and Monitoring Systems

The integration of technology into healthcare has revolutionized patient care, with health remote monitoring systems (HRMSs) emerging as powerful tools. By storing data, such as heart rate (HR) and oxygen saturation (SPO2) levels, HRMSs help medical professionals to treat patients with COPD. These systems offer real-time monitoring and personalized treatment options. However, to maximize the potential of HRMSs, it is crucial to integrate them with well-defined clinical processes, therapeutics, and rules. This integration ensures that the collected measurements are correlated and directly linked to effective patient care, enabling proactive interventions and improving health outcomes.

The Internet of Things plays a crucial and influential role in the successful implementation of HRMSs. Wearable device sensors, videos, and images are essential to gathering valuable patient information. Daily physiological data of the patient is collected and stored by the HRMS through data processing tools, analytics, and artificial intelligence (AI). Recording daily physiological data provides healthcare providers with actionable insights, facilitating proactive and personalized care.

The use of AI by HRMSs to predict patient health deterioration is a significant benefit. AI algorithms examine historical patient data to find patterns that might point to higher risks of unfavorable events or health deterioration. These forecasts offer healthcare professionals with insightful information that enables them to intervene early and prevent complications. A more preventive model of care is promoted by this proactive approach, which also enhances patient safety and lowers hospital admissions.

### 1.3. Effect of External Variables (Climate, Humidity, Particles) in COPD Patients

Over the past decades, several epidemiological studies have demonstrated the adverse health impacts of exposure to particulate matter (PM), both in coarse and fine fractions [4,5,6,7]. The origin of this particulate matter can be natural, such as desert dust, or anthropogenic, such as aerosols generated by biomass burning or fossil fuel combustion processes. The concentration of particles in the atmosphere depends on the emission sources, meteorological variables, and transport processes, as aerosols can travel long distances (transported by air masses). Additionally, house activities can be relevant sources of fine particles. Particles resulting from cooking and heating can more deeply enter the respiratory system, especially when they are finer.

The household air pollution data from the World Health Organization pointed out that among the 3.2 million deaths from household air pollution exposure, 19% are from COPD, and 23% of all deaths from COPD in adults in low- and middle-income countries are due to exposure to household air pollution [8].

### 1.4. Research Questions and Problems

The research question addressed by this study is: “Is it possible to automatically monitor and analyse the risk of potential health deteriorations of COPD patients?”. With this research question in mind, the defined objective is to develop a system that is capable of providing early information concerning patient health evolution and risk analysis in order to support the treatment of patients with COPD. Additionally, the system allows healthcare professionals to more efficiently manage their time by automatically providing said professionals with alerts, supported by a risk analysis of the patient’s COPD health status.

### 1.5. Purpose and Description of the Present Work

The Hope Care Intelligent Services Platform (HC PSI) is a P2020 project that involves the participation of Hope Care SA, INOV—INESC Inovação and the University of Beira Interior. Its main objective is to research and develop an intelligent services platform that enables healthcare professionals to make more informed decisions regarding the health conditions of COPD patients, thereby increasing the efficiency of clinical entities.

The components of the HC PSI include a CIDSS, HCAlert platform, and environmental data sources, all geared toward automating the clinical treatment of COPD patients who are being remotely monitored.

This research work focuses on the CIDSS developed by INOV—INESC Inovação. The CIDSS assists in making decisions regarding patient treatment. This platform is composed of three modules: an HRMS that provides patients’ health information through a mobile application to the CIDSS, a TVM that receives and processes patient risk information from the CIDSS, and a graphical user interface (GUI) that displays relevant clinical information to healthcare professionals.

**Figure 1:**
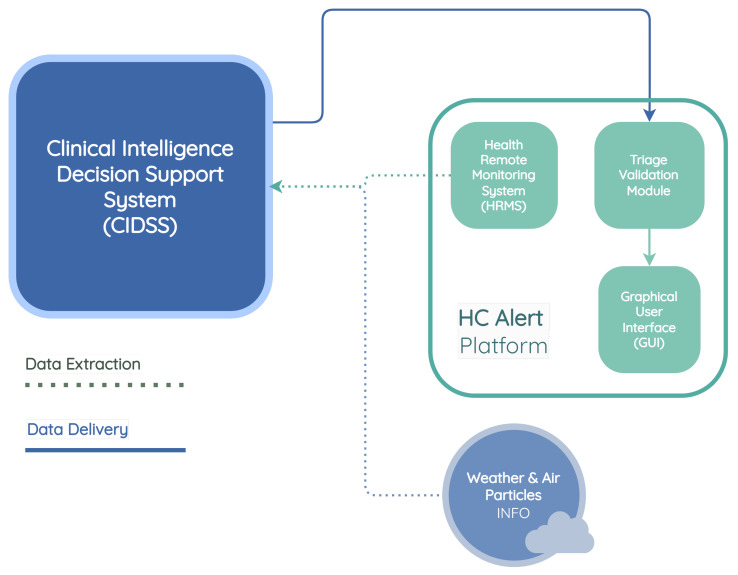
HC PSI architecture, including the CIDSS developed by INOV, the HCAlert platform, and other external data sources.

Figure 1 presents the HC PSI architecture, which includes the CIDSS developed by INOV, the HCAlert platform, and other external data sources.

The HCAlert platform was developed by Hope Care SA and includes a mobile application that supports HRMSs and a set of backend services for clinical validation and triage.

In the scope of the HC PSI project, the requirements for the HCAlert mobile application include the collection of patient symptoms and residential data. For the clinical validation and triage backend services, the following requirements are defined:

- Capability to categorize alerts.
- Capability to provide early warning scores and other relevant metrics of patients to healthcare professionals.
- Capability to obtain information about hospital visits internally or from other sources.
- Enabling the clinical team to have an overview of new alerts for each patient, including the client’s name, data type, and last measurement date.
- Allowing the clinical team to define what relevant health values to display on the dashboard.

### 1.6. Methodology

In this research work, since we focused on artifact development, we applied the design science research methodology.

The DSR methodology is a research methodology that is commonly used in the field of information systems; it focuses on the development and evaluation of innovative artifacts, which include cutting-edge framework prototypes, techniques, and algorithms that address present-day challenges. It consists of the following six phases: problem identification, definition of objectives, design and development, demonstration, evaluation, and communication. This methodology focuses on creating and evaluating artifacts based on their effectiveness, quality, and usefulness in addressing real-world problems [9].

**Figure 2:**
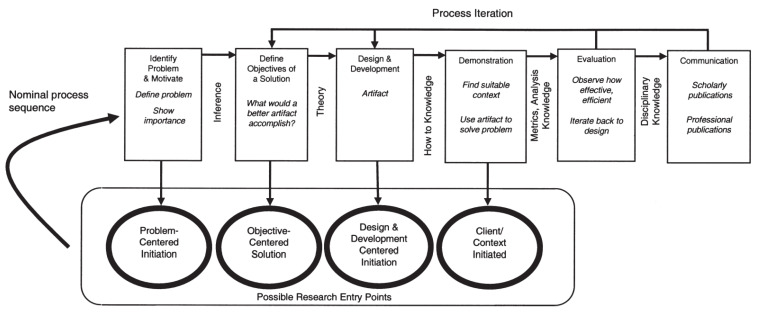
Iterations represented in the design science research methodology (DSRM) process model; Peffers et al. [10].

Figure 2 presents the iterations within the design science research methodology (DSRM) process.

## 2. Related Work

In this section, we present an overview of the systematic review conducted in this article, which follows the PRISMA (preferred reporting items for systematic reviews and meta-analysis) methodology [11]. This section covers the latest advances in managing pulmonary disease patients, particularly COPD patients. We emphasize the augmented efficacy that remote health monitoring brings to patient treatment by providing real-time warnings to medical professionals; we also discuss the enhanced effectiveness of remote health monitoring supported by predictive analytics, which provides early warnings about the risk of patient deterioration.

This systematic review also covers factors and biometric signs associated with acute deterioration in COPD patients and how the prediction of biometric signs and subsequent early warning generation can indicate the risk of future patient deterioration. Table 1 presents the topics and the respective queries used to extract and filter related works.

Table 2 presents the eligibility criteria used to filter documents in the related work.

**Figure 3:**
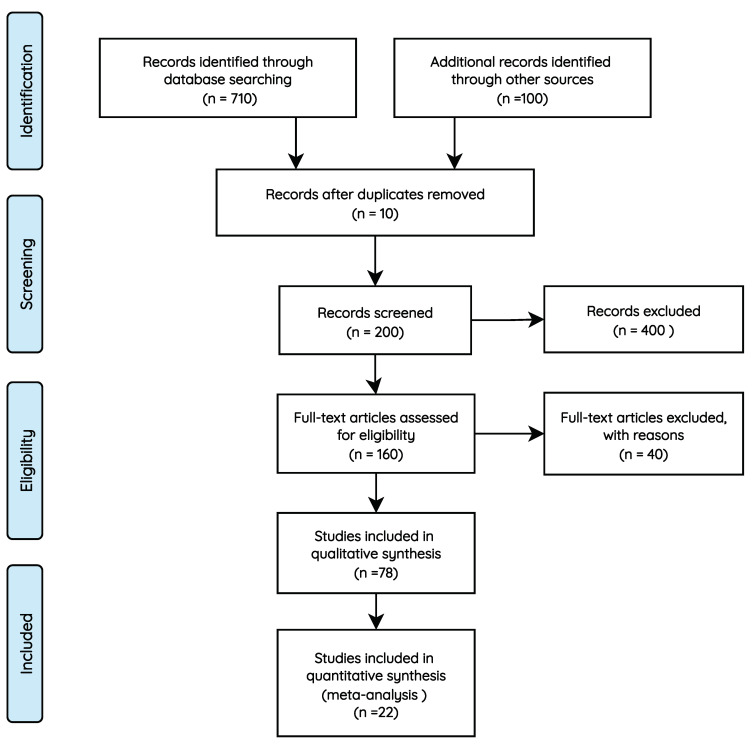
PRISMA methodology [11].

We identified 810 documents, with 10 documents removed due to duplication issues. A total of 400 articles not related to healthcare or artificial intelligence (AI) were excluded from further screening based on titles and abstracts. Moreover, 40 articles were excluded as we were unable to access their full versions, leaving 160 articles for full-text screening. A total of 82 articles were removed as they did not fit the eligibility criteria. Finally, 56 articles were excluded as they did not contain relevant information concerning vital signs, time series techniques, and health remote monitoring systems. The selection results, according to the PRISMA flow diagram, are shown in Figure 3.

### 2.1. In-Home Healthcare for COPD

Home telemonitoring is a term used to describe the utilization of audio, video, and other telecommunication technologies for monitoring a patient’s status from a distance [12]. This approach involves the remote monitoring of a patient’s health parameters, typically within the framework of a larger chronic care model. In fact, telemonitoring is an essential component of telehealth and telemedicine [13]; it has the potential to help patients manage disease and predict complications [14]. Telemonitoring projects involving patients with pulmonary conditions have demonstrated the ability to identify early changes in the patient’s condition, thus supporting immediate intervention and avoiding exacerbation. Patients have been very receptive to telemonitoring as a patient management approach and have shown very positive attitudes toward it [12]. A systematic review and meta-analysis found that telemonitoring interventions prevent unnecessary ER visits and may help to reduce severe COPD exacerbation to some extent. In 20 studies (90%) that carried out telemonitoring interventions for six months, a meta-analysis showed that the intervention effectively reduced the number of ER visits (pooled SMD = 0.14 corresponding to a small effect size; 95% CI (confidence interval): −0.28, −0.01) [13]. In a retrospective, population-based cohort study on 944 telemonitoring and 9838 control individuals, the total direct medical costs were significantly lower in the telemonitoring group (EUR −895.11, *p* = 0.04). The main driver for the total cost difference was the reduction in hospitalization costs by EUR −1056.04. (*p* = 0.01). A lower percentage of individuals died in the intervention group than in the control group (3.23 vs. 6.22%, *p* < 0.0001), translating into a mortality hazard ratio (HR) of 0.51 (95% CI: 0.30–0.86). Over the 12-month period, the proportion of patients hospitalized due to all causes (−15.16%, *p* < 0.0001), due to COPD (−20.27%, *p* < 0.0001), and for COPD-related emergency department (ED) visits (−17.00%, *p* < 0.0001) was consistently lower in telemonitoring patients, leading to fewer all-cause admissions (−0.21, *p* < 0.0001), fewer COPD-related admissions (−0.18, *p* < 0.0001), and fewer COPD-related ED admissions [15].

### 2.2. E-Healthcare Supported by Predictive Analytics

Telemonitoring has become indispensable in diagnosing and medically intervening for COPD patients. Nowadays, due to better storage of electronic health records and improved vital sign detection methods, large amounts of patient data are available daily in ICUs [16]. Medical equipment, ranging from hands-free monitors and portable devices to modern wristbands and watch-like monitors, have helped in the collection of biometric data, such as heart rate, blood pressure, physical activity, and sleep information [17].

A remote monitoring system, capable of gathering extensive data and backed by predictive analytics algorithms and techniques for effective data assessment and identifying underlying patterns, provides better efficiency in identifying declining patient health [18]. In the present COPD case study, such systems can reduce emergency room (ER) visits, acute deterioration-related readmissions, days spent in the hospital, and mortality in patients with COPD [19].

Predictive analytics refers to the systematic use of statistical or machine learning methods to make predictions and support decision-making. Predictive analytics applied to healthcare can be divided into two components: the data underlying the model, particularly predictors or features, and machine learning and statistical methods, both based on a set of mathematical techniques applied to data in order to generate an output [20].

Machine learning is a crucial methodology in predictive analytics. Conventional statistical analysis focuses on explaining data and relies on an expert (i.e., human) to formulate and discover cause–effect relationships, driven by a set of predefined assumptions. Machine learning is more data-focused and orientated toward generating hypotheses and building predictive models using algorithms. It has enabled clinical support research and applications to provide actionable insights by utilizing large amounts of intensive care unit patient datasets that are useful in many clinical scenarios [16]. Machine learning can predict in-hospital mortality and the risk of 30-day readmission due to COPD exacerbation [21].

### 2.3. Factors Associated with COPD Exacerbation

The prevention of acute exacerbation in COPD requires the identification of factors associated with exacerbation. Most studies have shown that oxygen saturation (SpO2) (*p*-value < 0.05), respiratory rate (RR), and heart rate (HR) (*p*-value < 0.05) influence exacerbation events, with SpO2 being the most predictive vital sign. The deterioration in COPD patients has been associated with a slight decrease in oxygen saturation and a slight increase in HR. One article suggested that using multiple vital signs as the inputs of a single classifier could provide better predictions, given that these multiple-input models showed the best AUC results [22].

Although some studies monitored blood pressure in order to determine whether there was a significant correlation with acute exacerbation, there was no sufficient evidence indicating that a change in blood pressure during a COPD exacerbation was a potent predictive factor for exacerbation (*p*-value > 0.05, i.e., not significant).

Body temperature with a *p*-value equal to 0.059 could be considered an exacerbation predictor. In the study conducted by Martin-Lesende, changes in body temperature had triggered 27.8% of alerts, of which, 5% were due to temperatures exceeding 37 ${}^{\circ }$C [23].

Most studies have focused on vital signs and internal factors of COPD patients, rather than external ones, despite being equally relevant. Some meteorological data, such as humidity (*p*-value = 0.0137), variation of diurnal temperature (*p*-value = 0.0472), the cumulative lowest temperature 7 days prior to acute deterioration (*p*-value = 0.005), and total rainfall in the 7 days preceding an acute exacerbation (*p*-value = 0.0389) was associated with acute exacerbation in COPD. Lee J. [24] conducted a univariate analysis of air pollution and COPD exacerbations and identified a strong correlation between PM10 levels one day before a patient’s condition worsened and acute exacerbation (*p*-value = 0.0260) [24].

The analysis of both internal and external factors with significant correlations to COPD exacerbation revealed that the frequency with which certain variables are measured must also be taken into consideration. The higher the frequency of a vital sign measurement, the better the perception of its association with an exacerbation occurrence. Daily or multi-daily vital sign monitoring improves the analysis of these signs. For example, Pépin J-L [17] mentions that overnight pulse oximetry increases sensitivity, allowing for early detection of deterioration [17].

### 2.4. Machine Learning for Early Identification of Deterioration

In recent literature, machine learning techniques have attracted attention for predicting the clinical conditions of patients. Time series forecasting models have been applied successfully in medical applications to predict disease progression, estimate mortality rates, and assess time-dependent risks. These models are able to identify patterns and trends from sequential data collected over time, such as health-related signals [25,26].

Some traditional machine learning techniques, such as random forest, SVM (support vector machine), Bayesian networks, and logistic regression, have been employed to improve predictive performance in identifying early clinical deterioration [27]. However, these traditional models are not optimized for handling the unique characteristics of time series data, such as autocorrelation, seasonality, and trend patterns [28,29].

With sufficient data, the development of deep learning models can reduce several preprocessing steps, emphasizing the relationships between the data, without the need to identify the best predictors, leading to better results [30]. For instance, long short-term memory network (LSTM) can learn extended time series dependencies, while a convolutional neural network can generate a compact latent representation.

Gradient boosting models are alternatives to specialized models, such as long short-term memory network (LSTM) and gated recurrent unit (GRU) [31,32]. Although these models are not ideal for time series forecasting, they are still generally better suited for handling sequential data compared to non-sequential algorithms (such as random forest, SVM, logistic regression, and naive Bayes) [29].

## 3. CIDSS Design

The CIDSS receives every patient’s vital signs, which are remotely monitored by Hope Care SA as inputs. Additionally, it daily incorporates weather forecast conditions and air particle forecasts that are specific to each patient’s location. In response, the system provides daily vital sign predictions and early warning scores for each patient for the following five days. It also provides the basal values of each patient and issues an alert whenever a vital sign measurement falls outside the expected parameter range, requiring a reevaluation.

**Figure 4:**
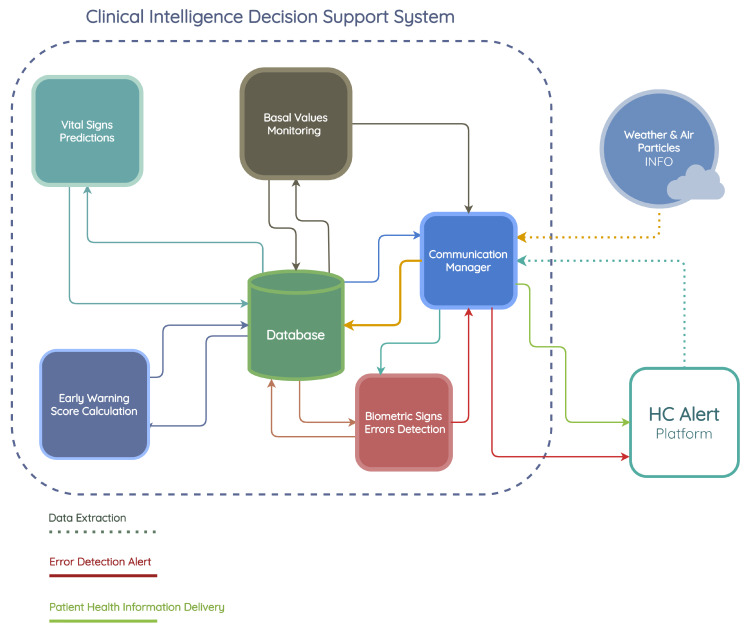
The CIDSS architecture and interactions with external modules.

Figure 4 illustrates the CIDSS developed by INOV—INESC Inovação, its interactions with weather and air pollution data providers, and the HCAlert platform. The CIDSS comprises five distinct modules, each serving a specific purpose. These modules are as follows:

Communication manager—this module assumes a crucial role within the system, and is responsible for the communication interactions among HC (Hope Care) Alert, weather, air particles API, and the clinical decision support system.

Vital sign prediction module—it is designed to generate forecasts for a five-day period regarding four essential vital signs: oxygen saturation level (SpO2), heart rate, systolic blood pressure (SBP), and body temperature. This module utilizes various machine learning algorithms to accomplish the predictions. The input data for these models are sourced from the stored vital sign records within the database. Subsequently, the predicted vital signs are stored back in the database for further reference and analysis.

Early warning score calculation module—within this module, the recorded vital sign predictions from the database play a crucial role in calculating the early warning score for each of the five predicted days. The early warning score is computed using the aforementioned vital sign data and the resulting early warning scores are subsequently stored in the database.

Biometric signal error detection module—the primary objective of this module is to thoroughly analyze and evaluate potential measurement errors and abnormal variations detected within the patient’s historical data. The purpose is to promptly alert both the patients themselves and the attending nurse regarding the invalidity or questionable nature of the entered information. By diligently identifying such anomalies, this module serves as a critical mechanism for ensuring data accuracy and reliability within the system.

Basal value monitoring module—the main function is to monitor and continuously and intelligently adjust the patient’s baseline values. This adjustment is based on the historical records of vital sign values measured by the patient and documented within the HCAlert platform. The module’s purpose is to enhance the precision and effectiveness of the monitoring system by dynamically adapting the baseline values in accordance with the patient’s specific health history.

### 3.1. Requirements

During the initial phase of the HC PSI project, we defined the functional requirements through an interactive and iterative process involving UBI and Hope Care SA. Certain clinical-oriented requirements were specifically delegated based on their domain of expertise. Subsequently, the remaining requirements served as the fundamental basis for the development of the CIDSS discussed in this article. All CIDSS functional requirements have been grouped into system modules, as shown in the following Table 3.

### 3.2. Communication Manager

**Figure 5:**
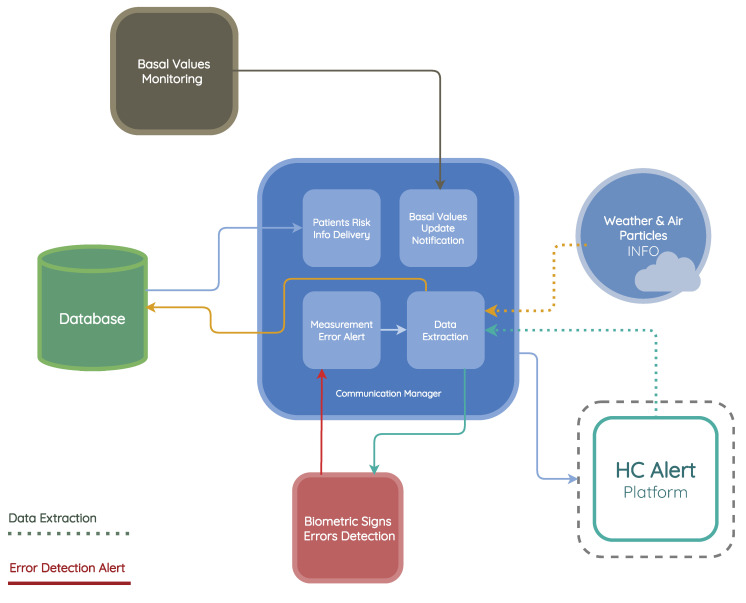
Communication manager module architecture.

This module is composed of four submodules: data extraction, measurement error alert, basal values notification, and the patient’s risk information delivery submodule, as is present in Figure 5.

#### 3.2.1. Data Extraction

The medical records, which stored the vital signs used as input for the CIDSS, are presented in Table 4. Each record is formatted to have one entry per day per parameter. Each record had an ID (idRawMeasurement), the collection date (createdOn), the coordinates where it was collected (latitude and longitude), the measurement type (ProviderMNameStandard), measurement value (value), and the units representing the value (units).

The measurement type could address various factors, including vital signs, such as oxygen saturation level (SpO2), heart rate (HR), body temperature, systolic blood pressure (SBP), and diastolic blood pressure (DBP), as well as other biometric indicators, like the number of steps, body fat, energy burned, weight, and height.

The weather historical information used as input for the predictive models was provided by the Weatherbit API. Each record had an ID (idWeatherMeasurement), the coordinates of the station (latitude, longitude), date of measurement (columns year, month, day), mean daily temperature (T_MED), and mean relative humidity (HR_MED), as shown in Table 5.

The air pollution historical information used as input for the predictive models was provided by the OpenWeather API. Each record had an ID (idWeatherMeasurement), the coordinates of the station (latitude, longitude), date of the measurement, an average count of 10-micrometer particles (PM10), and an average count of 2.5-micrometer particles (PM2_5), as shown in Table 6.

#### 3.2.2. Measurement Error Alert

This submodule was designed to receive alerts from the biometric sign error detection module and subsequently send alerts to the HCAlert platform. After a set short duration, it sends a notification to the data extraction submodule to execute the data extraction of biometric signs from HCAlert, concerning the specific patient dataset where the error was found.

#### 3.2.3. Basal Value Monitoring Notification

The basal value update notification submodule was designed to receive notifications from the basal value monitoring module; it subsequently notifies the HCAlert platform with new basal value recommendations for a specific patient.

#### 3.2.4. Patient Risk Information Delivery

The patient risk information delivery submodule extracts information regarding the last five days of vital sign predictions and the calculated early warning scores stored in the database. It then sends this information to the HCAlert platform.

### 3.3. Biometric Sign Error Detection

The HCAlert platform’s operational efficiency is affected by the patients’ inaccurate vital sign measurements, which can result in inaccurate clinical protocol adjustment alerts and future vital sign projections. It is necessary to guarantee that the system receives data that obey certain quality levels.

Prior to the implementation of the current project, measurements are validated by nurses who identified instances of anomalous readings, reporting potential causes, such as deterioration in the patient’s condition, measurement errors, cold fingers during measurements, etc.

The biometric sign error detection module consists of three components:

- Validation of clinical rules: This component compares the measurements taken by the patient with a set of business rules defined according to Hope Care guidelines. For example, a measurement of oxygen saturation above 100 or below 20 cannot be correct since a percentage value cannot exceed 100, and a value below 20 corresponds to situations of compromised brain function and even comas. The medical team involved in this research work validated all ranges used to filter the vital signs.
- Patient pattern modeling: The objective of this component is to approximate a probability density function for each metric in the patient’s measurements. These probability density models are then stored in the database, eliminating the need to repeat the function modeling each time a new inference is made. This module runs monthly to create a new probability function that captures the variability of the new measurements entered by the patient.
- Validation of atypical measurements based on the patient’s history: This module uses the probability density models stored in the database, which are associated with each patient’s vital signs, to determine whether a newly recorded measurement falls within the normal patterns for that specific patient. As these variations could be due to disease exacerbation, improvements from a new medication, or other factors, need to be validated by a nurse and, if necessary, by the patients themselves, to determine the true cause of the variation.

**Figure 6:**
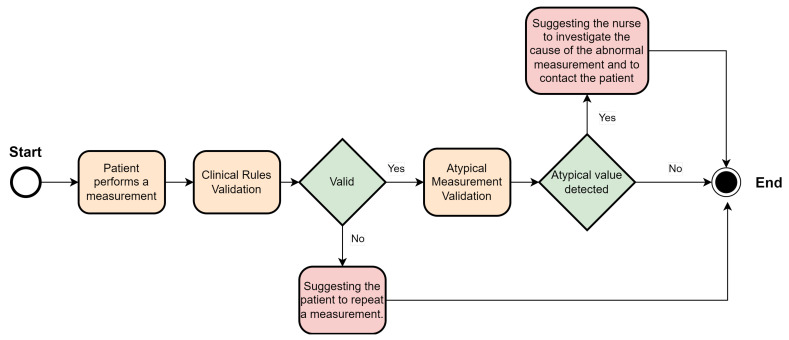
Biometric sign error detection implementation.

The operationalization of this module is presented in Figure 6. The system begins with the measurement and input of a vital signal by a patient in the HCAlert application. The measurement is compared and validated based on clinical rules, according to the type of measurement performed. The following clinical rules are defined, where the value is considered erroneous and discarded in the following cases:

- Oxygen saturation above 100 or below 20;
- Body temperature below 30 or above 40;
- Systolic blood pressure below 50 or above 350;
- Diastolic blood pressure below 40 or above 200;
- Pulse rate less than or equal to 30, or greater than 250.

**Figure 7:**
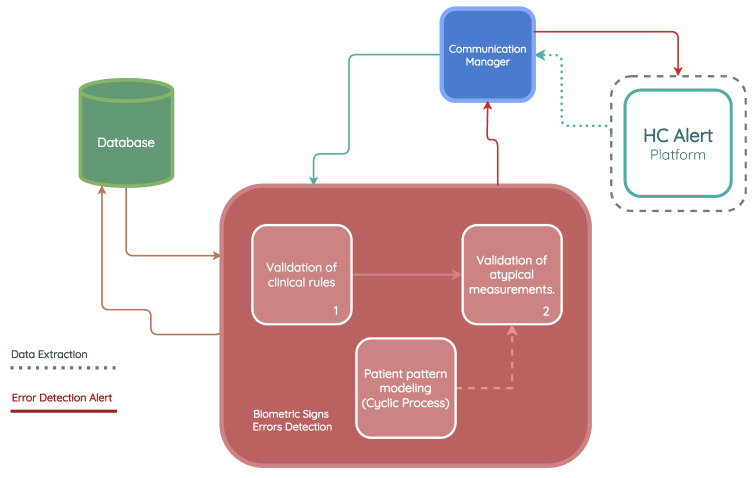
Biometric sign error detection module architecture.

Figure 7 presents the architecture of the Biometric sign error detection module.

In the event of an incorrect measurement, a type 1 alert is triggered, recommending a new measurement of the vital signal by the patient.

If there is no inconsistency with the rules, the system then determines if the measurement is atypical for a patient. If it is not considered atypical, the verification process is concluded without any identified errors. If an atypical value is recorded, a type 2 alert is triggered, and human verification of this alert is recommended to a nurse and the patient. This is done to verify whether this value corresponds to a health deterioration, an improvement in the clinical condition, or a measurement error.

**Figure 8:**
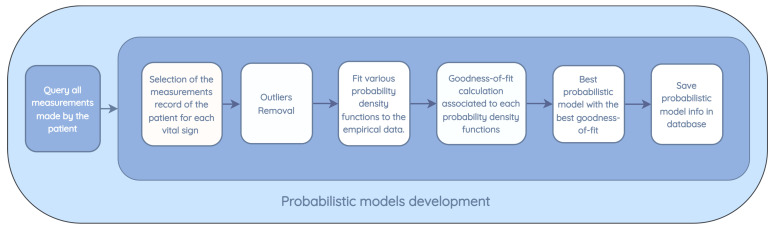
Biometric sign error detection model development.

Probability density functions were applied in order to model the pattern of vital signs of each patient and assess the probability that a newly measured value fits the distribution function computed for that specific patient’s vital sign. The process of training a model for a given patient begins with the request for all the vital sign measurements made by this patient. From this request, as shown in Figure 8, a distribution function is trained and stored in the database for each vital sign recorded, with the following steps:

1. From all the measurements collected for the patient, only the measurements made for specific vital signs in training are used.
2. Existing outliers in the database, prior to modeling, are removed. Outliers are removed based on the standard deviation by calculating the standard score (z-score), which corresponds to the number of standard deviations by which a newly recorded value deviates from the mean of the observed measurements. If the z-score is greater than 3, which corresponds to a value that is three times the standard deviation away from the mean of the data, the value is not used in the modeling.
3. The following distributions are tested: normal, exponential, Pareto, double Weibull, t, generalized extreme value distribution, gamma, lognormal, beta, and uniform. For each distribution, the density and weights of the histogram are computed. Subsequently, an estimation of the function parameters is performed based on the data. The maximum likelihood estimation (MLE) is used to identify the values that best fit the data.
4. The goodness-of-fit is calculated with a test of the sum of squares of the residuals for each distribution found.
5. The model with the best goodness-of-fit, which implies a lower value in the sum of squares of the residuals, is stored for the vital signs of the patient under study.

**Figure 9:**
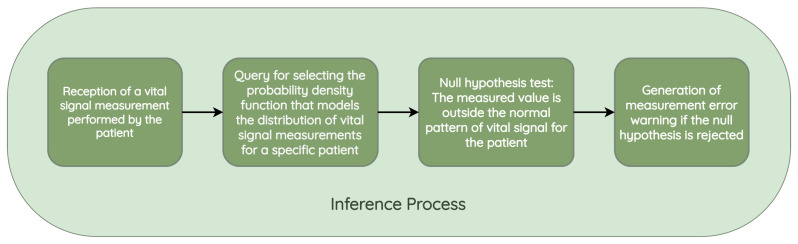
Biometric sign error detection inference process.

The inference starts with the reception of a vital sign measurement taken by a patient and entered into the HCAlert system. The system selects the model corresponding to the probability density function that models the distribution of the vital signs measured for the patient who entered it into the system, as is present in Figure 9.

This model is then used to test the null hypothesis, which corresponds to checking whether the value that has been measured is outside the typical pattern of the patient, based on the selected distribution and the parameters adjusted according to the empirical distribution of the patient. If the *p*-value is less than 0.05, it implies that the null hypothesis is not rejected, which means that there is a probability that the measurement may correspond to an error, exacerbation, or improvement of the condition. A reminder should be sent to both the nurse and the patient to investigate the situation.

### 3.4. Basal Value Monitoring

The deterioration or improvement of COPD reflected in the negative or positive evolution of the patient’s baseline values may be due to several explanatory factors, such as weather conditions, exposure to particulate matter, a change in medication or lifestyle, among others. The recorded baseline values are indicative of the severity of a condition, as outlined by the Global Initiative for Chronic Obstructive Lung Disease (GOLD) [33] strategy for the diagnosis, management, and prevention of COPD.

Values below or above the standards result in the patient’s category changing into one of the GOLD I–GOLD IV [33] categories, depending on the severity of the patient’s condition, with GOLD I being the most severe condition. It is important to identify and monitor any deterioration in a patient’s baseline values in order to adjust the clinical protocol and treatment guidelines.

**Figure 10:**
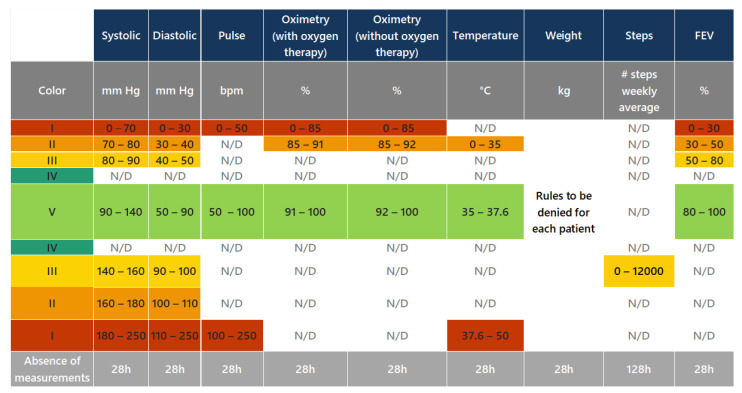
Clinical protocol defined by the Hope Care SA Medical Team and based on the GOLD clinical protocols.

Figure 10 presents a clinical protocol defined by the Hope Care SA medical team; it is based on the GOLD strategy and addresses patients whose basal values are within a normal range and, thus, do not belong to categories GOLD I–GOLD IV. Consequently, the range of colors isn’t associated with the GOLD categories. The color is associated with the severity of the COPD patient’s condition: Category I (red) corresponds to a higher degree of deterioration in their health condition, while Category V (green) corresponds to the lowest or non-deterioration of their health condition. Some fields are filled with the expression “N/D” because there is no defined range of values for that specific category.

#### 3.4.1. Basal Value Monitoring Module Architecture

**Figure 11:**
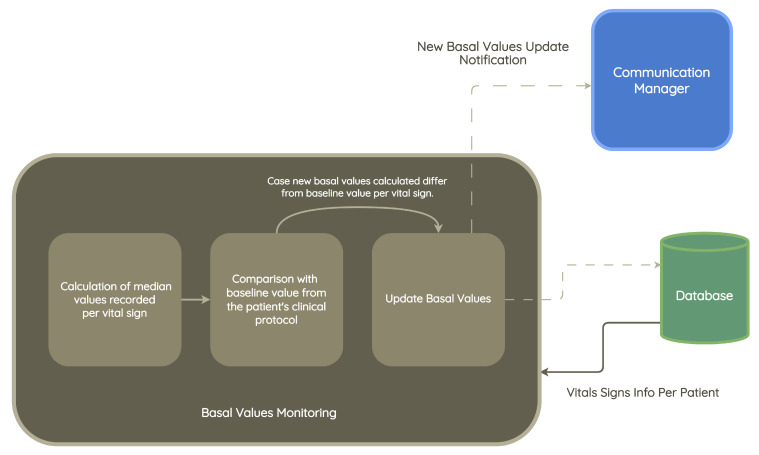
Basal value monitoring module architecture.

This module, as shown in Figure 11, uses the list of metrics to be monitored and the history of vital signs recorded by each patient as input. Based on these measurements, the patient’s current baseline value and the forecast of the evolution of the same value are determined. In case there is a substantial difference between the most recently recorded value and the historical baseline value, an alert should be triggered, containing the previous baseline value, the newly calculated value, and the difference. The newly calculated baseline value is suggested as a change to the clinical protocol.

The following variables are also used as input to the module:

- Number of months considered: This indicates the past time window that is analyzed for the baseline calculation. The default value is 3 months, which indicates that when this module runs, the measurements taken from the last 3 months are extracted for the baseline calculation. This value can be configured by rules in the system.
- Minimum number of records: This corresponds to the minimum number of measurements taken by the patient, so that the calculated baseline information is considered reliable. If the patient does not have a satisfactory number of measurements in the time horizon under study, the module will not provide recommendations. For example, a patient with only five SpO2 measurements over 3 months will not be considered for updating the baseline value. This value is configurable by a rule, and value 50 is used by default in the system.
- Patience: In case the patient does not present enough measurements of a certain parameter in the defined time horizon, the system expands the time horizon of the search to include more months of history until it finds an acceptable amount of records. For example, with a patience of 3 months and a minimum of 50 required measurements, if the patient only has 30 measurements, an additional month will be incorporated into the analysis, and the module will be rerun using the past four months, reducing the patience counter by 1. In case patience reaches zero, and the minimum value of measurements defined is not reached, the system will not provide any recommendation for the given parameter due to the lack of consistency in the measurements. The default value for patience, which can be configurable by a rule, is 3.

The default values in the system are set and adjusted after testing with historical values recorded by patients in the HCAlert platform, provided by Hope Care SA.

#### 3.4.2. Basal Value Monitoring Module Implementation

**Figure 12:**
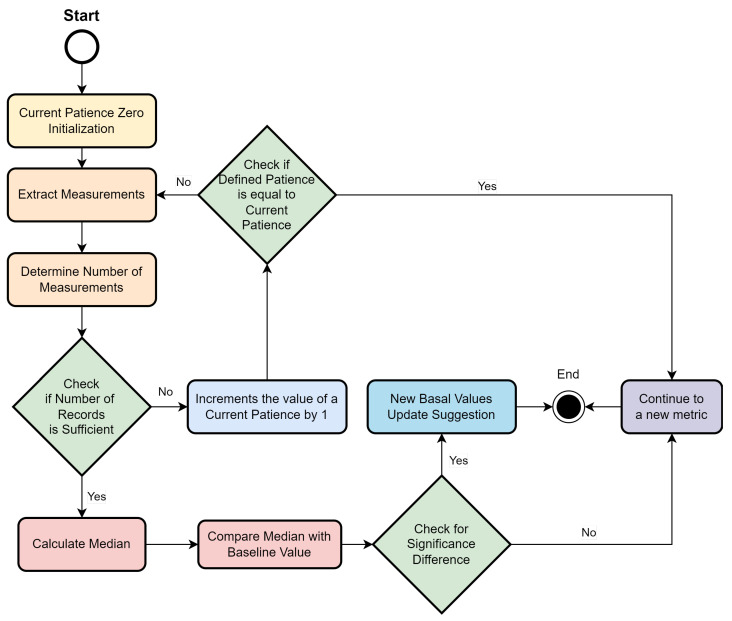
Basal value monitoring module implementation.

In this section, we present the implementation details of the basal value monitoring module. Figure 12 shows an activity diagram, which represents the operations performed by the module.

As presented and detailed in the previous section, the system inputs are the list of metrics under evaluation, the patient’s vital signs history, the number of months to be considered, the minimum number of records, the baseline value of the patient’s clinical protocol, and patience.

For each metric under evaluation, the system performs the following process:

1. A flag representing the current patience is initialized to zero.
2. The measurements are related to the period of months corresponding to the last X months from the date of execution of the module, where X is the sum between the system input “number of months to consider” and the current patience value.
3. The number of measurements performed by the patient is calculated.
  (a) In case the number of measurements is not sufficient, the current patience is incremented by 1.
    (i) If the current patience value is equal to the user-defined patience value, no recommendation is displayed, and the cycle continues to the next measurement in the list.
    (ii) If the current patience value is less than the set patience value, the system summarizes the run from step 2.
  (b) In case the measurements are sufficient, the system summarizes the run in step 4.
4. The median of the patient’s measured values of a given vital sign is calculated.
5. The median value is compared with the baseline value recorded in the clinical protocol.
  (a) If the values are very different, a recommendation is made to update the baseline value to reflect the new median value recorded in the time interval under consideration. This recommendation should be evaluated by a medical professional.
  (b) If the values are similar, the baseline value is not adjusted, and the system summarizes in step 1, with a new iteration of a new metric under evaluation.
6. The cycle ends when all metrics in the list have been processed.

This process is run independently for each patient in the system. It is worth noting the use of the median as the metric calculated for the baseline value. This is due to the fact that it better handles extreme values outside of a patient’s normal patterns, such as exacerbation, which should not be considered for the calculation of a baseline value, as it does not correspond to a normal patient pattern.

### 3.5. Vital Signs Prediction Module

#### 3.5.1. Predictive Model Development

##### Data Treatment

For the predictive model development and evaluation, 91 patients who were flagged as having COPD were included. Each patient was monitored remotely and provided health status information for tracking their health status. The vital sign information was then gathered by each medical center. These patients were from different districts of the country, such as Aveiro (Anadia), Leiria (Óbidos, Pombal), Santarém (Ourém) Castelo Branco (Fundão), Coimbra (Cantanhede, Cernache, Assafarge, Antanhol, Condeixa-A-Nova, Mira, Almargem Bispo), Lisboa (Amadora, Rinchoa, Queluz, Algueirão, Tapada Das Merces, Rio de Mouro), and Faro (Quarteira, Albufeira, Tavira, Olhão, Loulé, Lagos, Portimão, and Castro Marim).

Meteorological variables (temperature, humidity, wind, and rain) and exterior particle matter concentrations (PM10, PM2.5) were obtained from the nearest IPMA and EPA stations. To analyze the source and transport pathways of the air masses and relate the air masses with aerosols, we used the NOAA HYSPLIT model [34,35].

**Figure 13:**
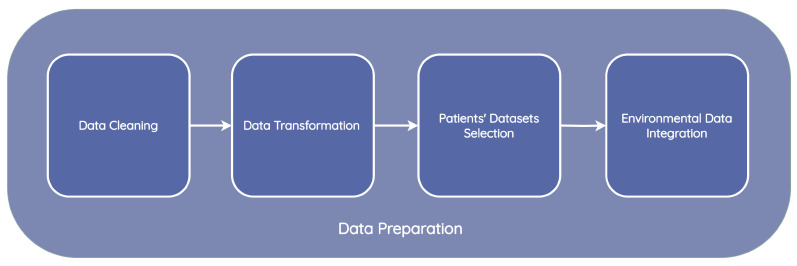
Data preparation pipeline.

Information about the weather, air quality, and vital signs was analyzed. The data processing module was divided into four sub-phases: data cleaning, data transformation, patient datasets selection, and environmental data integration, as is present in Figure 13.

**Figure 14:**
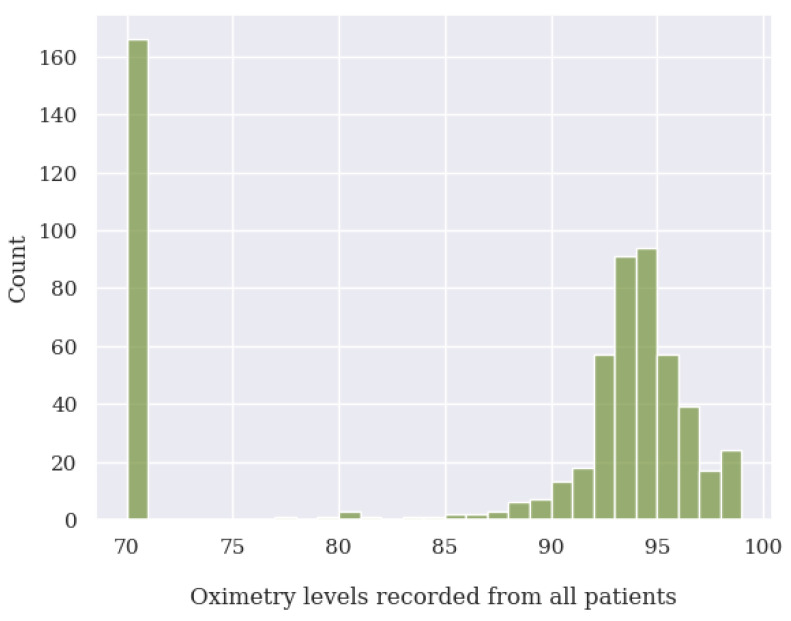
Oxygen saturation level value distribution of all patients analyzed.

**Figure 15:**
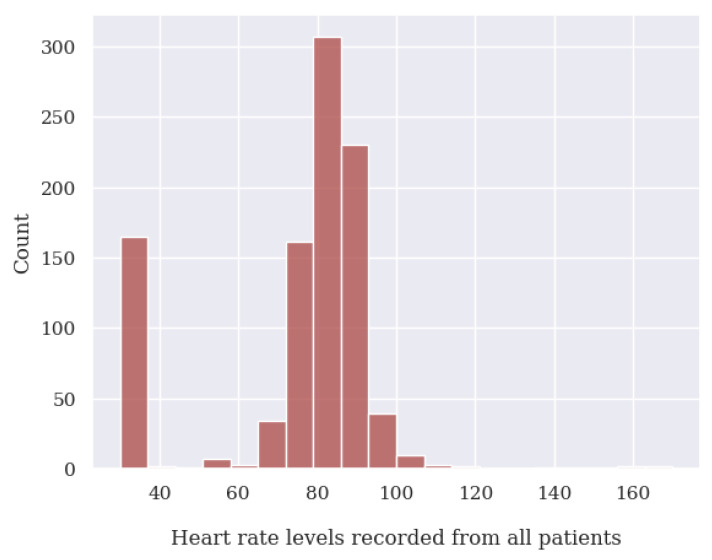
Heart rate level value distribution of all patients analyzed.

**Figure 16:**
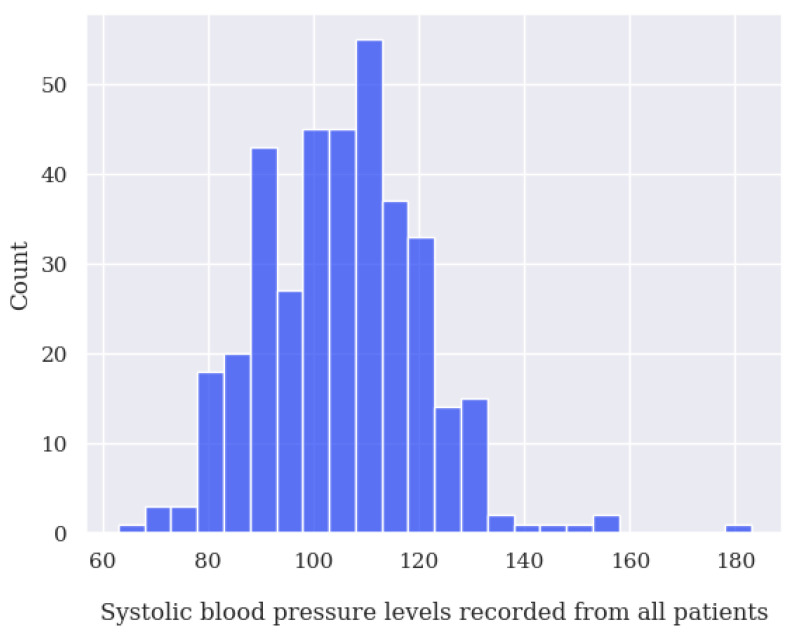
Systolic blood pressure value distribution of all patients analyzed.

**Figure 17:**
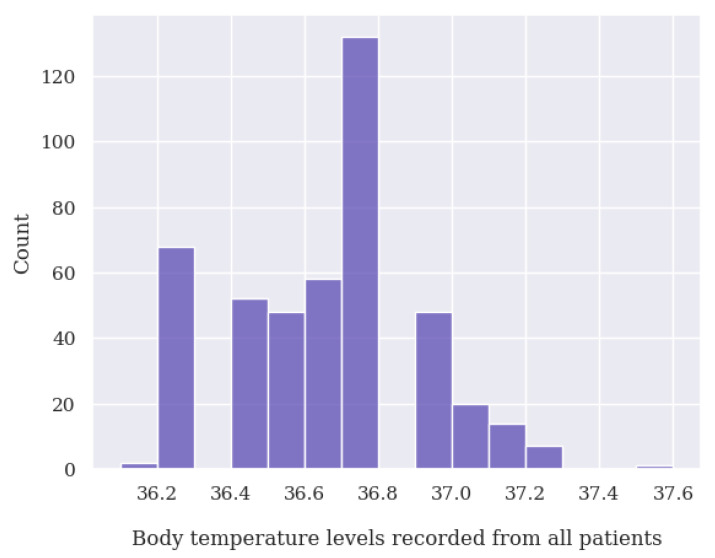
Body temperature value distribution of all patients analyzed.

During the data cleaning process, a thorough analysis was conducted on outliers (values that deviated significantly from the rest of the dataset and could potentially introduce anomalies in the results obtained from algorithms and analysis systems) based on the distribution of values in Figure 14, Figure 15, Figure 16 and Figure 17, as well as on null values within the vital signs.

Regarding vital signs, any values that met the following criteria were identified as outliers and subsequently removed:

- For oxygen saturation (SpO2), any values below or equal to 70% and above 100%. Since we have detected many measurements at exactly 70%, we suspect these are measurement errors;
- For body temperature, all values below 30 ${}^{\circ }$C and above 40 ${}^{\circ }$C;
- For systolic blood pressure (SBP), any values below 50 mmHg and above 350 mmHg;
- For heart rate (HR), any values below 39 BPM and above 250 BPM.
- For diastolic blood pressure (DBP), any values below 40 mmHg or above 200 mmHg.

In the data transformation process, we adjusted the format of historical records related to the vital sign data of patients. The data, initially in a format of one record per day per parameter, were converted to one record per day with all the collected vital sign values for that day. Specifically, there was a change in the granularity of each data row from one row per measurement of a specific vital sign at a specific moment in time for a specific patient to one row for each day of measurements taken for a specific patient, with columns representing the measured vital signs (data pivoting). After the format change, every time segment with over 10 consecutive days of missing data was removed and only patients with over 180 records whose vital sign data were fully complete were selected.

In the data integration process, the historical records of each patient’s vital signs were supplemented with information regarding weather data (average daily temperature, average relative humidity, and amount of daily precipitation) and air particle data (10 µm particles and 2.5 µm particles, as these two dimensions have a greater impact on the patients’ respiratory capacity).

##### Modeling and Evaluation

**Figure 18:**
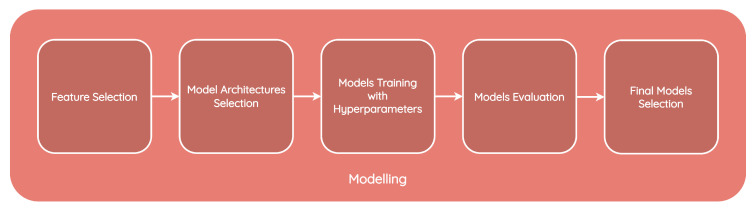
Modeling and evaluation pipeline.

Following the data treatment, we modeled the development and evaluation. As a result of the data treatment phase, only 14 datasets were considered for the model training and evaluation phase. Since the CIDSS was designed to assist COPD patients with different health profiles, we developed models using 14 different datasets and incorporated the best models in the system. Figure 18 shows the steps of the development and evaluation phase.

We employed multivariate machine learning models capable of conducting the multi-step-ahead time series prediction of vital signs. Multi-step-ahead forecasting involves predicting multiple future time steps in a time series [36]. In our case, it would mean predicting the vital sign values for the following 5 days. The vital signs chosen for prediction include SpO2, heart rate, body temperature, and systolic blood pressure, which are utilized in the early warning score calculation module to assess the risk of deterioration.

During the feature selection process, we conducted a comprehensive correlation analysis between vital signs and clinical validation, resulting in the identification of the most relevant vital signs for predicting health variations in COPD patients.

**Figure 19:**
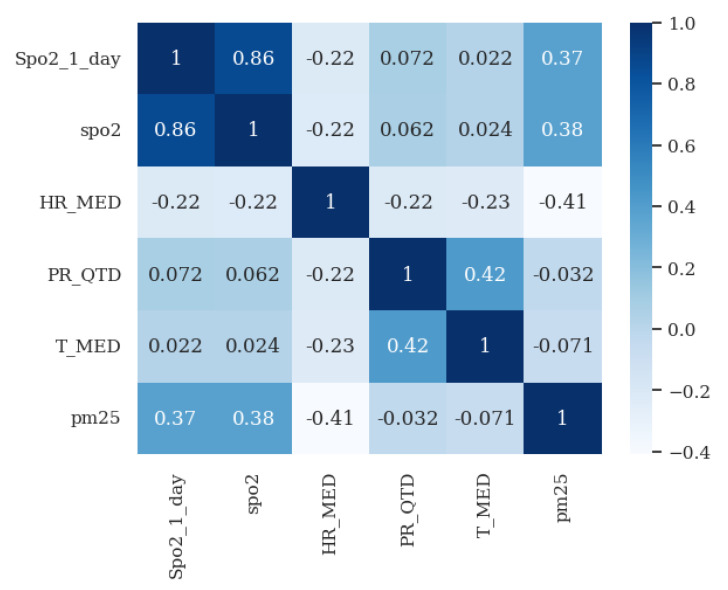
Correlation matrix of values of the SpO2 parameter with the relative humidity, the levels of precipitation, the pm25 concentration, the external temperature values, and SpO2 level from the previous day, using the dataset for the patient with ID no. 156.

Figure 19 shows an example of a correlation between SpO2 values (Spo2_1_day), the pm25 external parameter (PM25), relative humidity (HR_MED), and SpO2 values (SpO2) of the previous day, using the dataset for the patient with ID no. 156.

For multi-step-ahead time series prediction, all vital signs receive the previous day’s value (n − 1) as input to forecast the value for the current day (n). To predict the value of SpO2, we selected the following inputs: the SpO2 value of the previous day, the relative humidity value of the previous day, the levels of precipitation from the previous day, the pm25 value from the previous day, and the external temperature value from the previous day.

**Figure 20:**
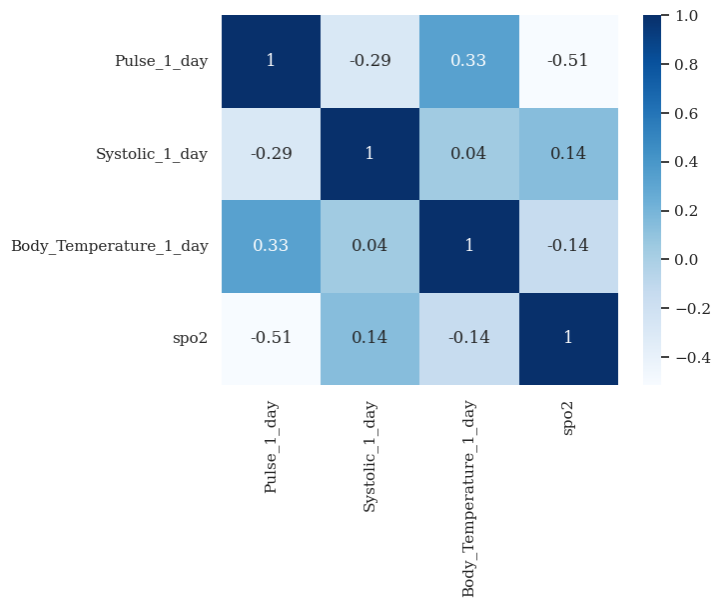
Correlation matrix of values of SpO2 parameter with the pulse rate, systolic blood pressure and body temperature values of the following day, using the dataset for the patient with ID no. 156.

Regarding the other vital signs, based on the analysis of the correlation between the four vital signs analyzed in Figure 20, and the clinical insight provided by the Hope Care SA medical team suggesting that SpO2 influences heart rate, body temperature, and systolic blood pressure, we decided to use only the SpO2 value from the previous day and the specific vital sign in question from the previous day as inputs.

To ensure the selection of the most optimal model architecture for predicting a specific vital sign, we trained and evaluated six distinct machine learning models. These models encompassed a diverse range of architectures, namely ARIMA (autoregressive integrated moving average), LSTM (long short-term memory), BILSTM (bidirectional long short-term memory), GRU (gated recurrent unit), LightGBM (light gradient boosting machine), and XGBoost (extreme gradient boosting).

The training process was preceded by essential hyperparameter tuning, which is a critical step in developing machine learning models. This tuning allowed us to optimize the models for the best possible performance. In our case, the models’ performance was assessed using the root mean square error (RMSE), which measures the difference between prediction and the ground truth in the regression algorithm evaluation.

Table 7 presents an example of the RMSEs achieved for the fifth-day predictions via different machine learning model architectures for each vital sign prediction using the dataset for the patient with ID no. 156.

As a result of our evaluation, we saved the models that demonstrated the lowest root mean square error (RMSE) for each vital sign. Consequently, we had 4 distinct models for each of the 14 patient-specific datasets, with each model specialized in predicting a specific vital sign.

Table 8 presents an example of the RMSEs for the 5th-day predictions achieved by the best machine learning model architectures for each vital sign prediction using the dataset for the patient with ID no. 156.

#### 3.5.2. Production

In this section, we present the incorporation of the previously described predictive models into the clinical information decision support system (CIDSS).

**Figure 21:**
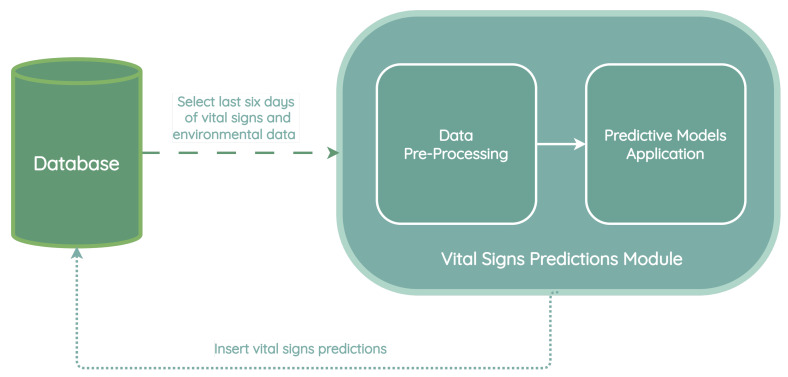
Vital signs prediction module architecture.

The vital signs prediction module presented in Figure 21 is composed of two sub-processes: a data pre-processing stage followed by the application of predictive models. The data pre-processing stage is essential to ensure that the data are in the correct format and that the vital sign measurements are appropriately integrated with the external measurements, as previously mentioned in Section 3.5.1.

The vital signs prediction process takes place daily, and the resulting predictions are stored in the database for future reference. Subsequently, the early warning module utilizes these data to assess and calculate the risk of a patient experiencing deterioration within the following five days.

When a new patient is integrated into the system, the prediction for each vital sign is calculated as the average of the predictions from all the models that predict the particular vital sign. After a period of 6 months, the error (root mean squared error—RMSE) of each predictive model is analyzed by measuring the distance between the values predicted by each model and the actual values of the vital signs for each patient. The model with the lowest error is the one associated with the patient.

### 3.6. Early Warning Score Calculation Module

In this module, the risk of a patient experiencing deterioration is assessed using the early warning score (EWS) clinical protocol. The EWS is utilized for monitoring and detecting the risk of health deterioration in patients and it is calculated by combining vital signs and clinical data, such as heart rate, blood pressure, respiration rate, body temperature, oxygen saturation (SpO2), and degree of consciousness. Individual scores for each vital sign are then totaled up, resulting in a total EWS score.

The higher the overall EWS score, the more likely a patient is suffering from a health deterioration. This clinical protocol presented in Table 9 is indicated by Hope Care SA’s medical team.

Similar to the vital signs prediction module, the early warning score calculation is performed daily, and the resulting scores are stored in the database.

## 4. Demonstration and Evaluation

To demonstrate how the CIDSS addresses the research question, we present a system trial with the incorporation of a new patient. We use the patient with ID no. 300. The patient health information used in this trial consists of historical information for a three-year period consisting of HRMS monitoring provided by Hope Care SA through the HCAlert platform.

The monitoring for the patient with ID no. 300 was initiated on 21 April, 2022. The CIDSS received a notification from the HCAlert platform, regarding the need to incorporate this new patient, leading to the creation of a new record in the database. All vital signs monitored for the patient with ID no. 300 were transmitted to the HCAlert platform and subsequently extracted by the CIDSS, starting from 21 April. These vital signs underwent analysis through the biometric sign error detection module. As no outliers were detected in the vital signs, they were seamlessly integrated into the database.

Table 10 presents the last five days of data extracted from the database for vital sign predictions on 25 April.

By 25 April 2022, a sufficient amount of vital sign data is available to provide insights into the patient’s risk of health deterioration. The CIDSS proceeds with the prediction of vital signs and subsequently calculates the early warning score. Various models are employed to forecast the patient’s vital signs for the initial 6 months of integration. The risk information regarding the patient’s potential deterioration is provided to the HCAlert platform through a JSON file.

Table 11 presents the vital sign prediction values for 26 April. The predicted vital signs are then used to calculate the risk.

Table 12 presents the values of the early warning score calculated on 25 April.

Listing 1 presents part of the structure of a part of the JSON file concerning the predicted vital signs and early warning score calculated from 26 April to 30 April.

**Listing 1.** Structure of the JSON file provided to HCAlert for patient risk information on 25 April.

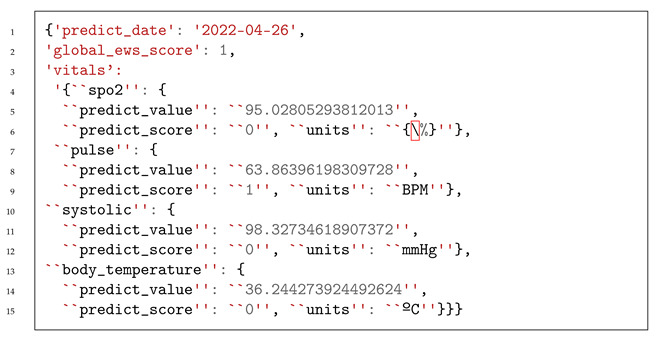



After an evaluation spanning over 6 months, we focused on identifying the most suitable models to enhance the care of patient 300. Our selection process prioritized models with the lowest root mean square error (RMSE), as shown in Table 13.

We analyzed the patient’s data from the previous 6 months; we provide a new basal value that reflects the patient’s health condition, which is, consequently, used for the patient’s clinical protocol adjustment, as shown in Listing 2.

**Listing 2.** Suggested new basal values for patient 300 to the HCAlert platform.

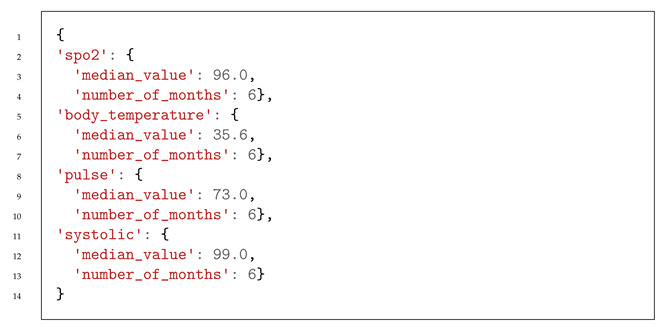



During the course of 6 months, while closely monitoring patient 300’s health, we detected an error involving one of the SpO2 measurements. Initially, this measurement seemed to comply with the clinical rules and was considered valid. However, upon atypical measurement validation, it became evident that the probability of this value (*p* = 0.01599) belonging to the distribution of SpO2 values for patient 300 was relatively low, falling below the threshold of 0.05. Due to this fact, this measurement was discarded from the dataset.

**Figure 22:**
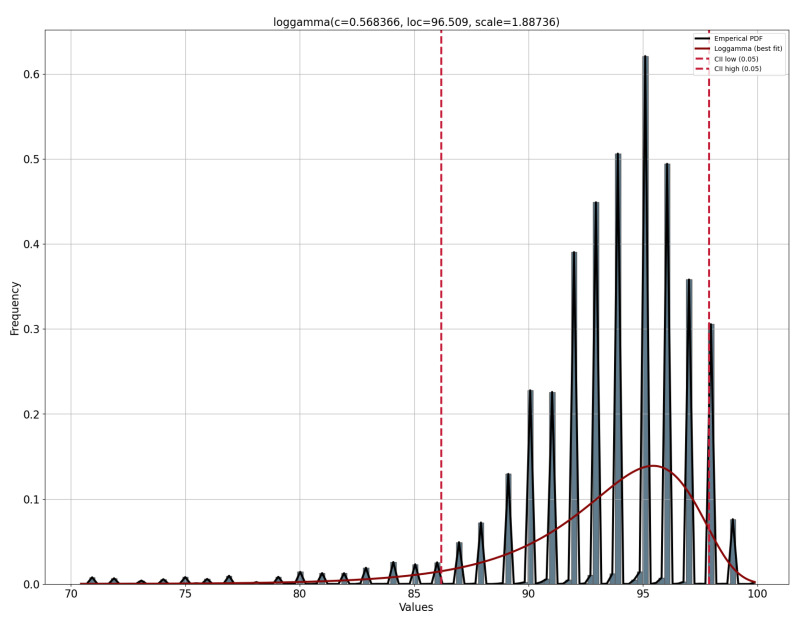
Distribution of SpO2 values analyzed of patient 300.

Figure 22 presents the distribution of SpO2 values of patient 300 analyzed for the error alert validation.

On 25 October, the CIDSS provided essential health information about the risk of patient deterioration. However, this risk was generated using predictions from the selected best models, as mentioned earlier.

Table 14 presents the last five days of extracted data from the database for vital sign predictions on 25 October.

Table 15 presents the vital sign prediction values from 25 October. The predicted vital signs are then used to calculate the risk.

Table 16 presents the early warning score values calculated on 25 October.

Listing 3 presents the structure of a JSON file concerning the predicted vital signs and early warning score calculated from 26 October to 30 October.

**Listing 3.** Structure of the JSON file provided to HCAlert for patient risk information on 25 October.

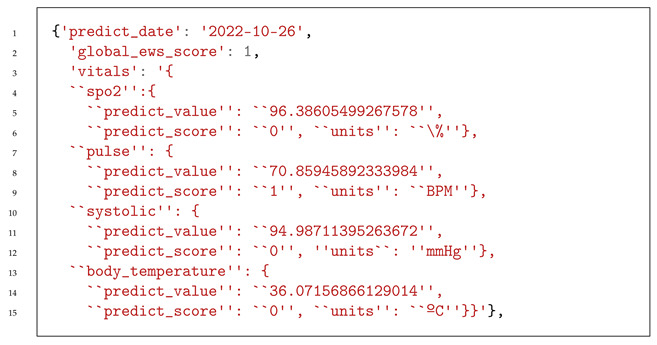



### System Evaluation

We performed a set of white-box tests, evaluating each module for its functionality (unit tests) and integration with the related modules of the system (integrated tests). Afterward, we conducted a survey to gather feedback from the health professionals to evaluate the system based on a set of criteria inspired by Prat et al. [37]. Based on the positive feedback collected from the survey, it appears that the system was well-designed and valuable for managing the treatment of COPD patients.

Table 17 shows the evaluation given by the health professionals. They were asked to answer questions, indicating a number between 1 and 5, where 1 corresponds to not relevant or not useful and 5 corresponds to very relevant or very useful.

## 5. Conclusions

### 5.1. Work Conclusions

In this paper, we developed a system prototype that answers our research question: “Is it possible to automatically monitor and analyse the risk of a potential health deterioration of COPD patients?”. This system aims to provide early information concerning a patients health status evolution in order to support the treatment of patients with COPD.

As mentioned in Section 3, the CIDSS comprises two primary components: the vital signs prediction module and the early warning score calculation module. These components specifically address the research question.

The vital signs prediction module, as mentioned in Section 3.5, generates vital sign predictions using different types of model architectures. These predictive models are optimized using a fine-tuning process, with each model corresponding to a specific patient with a specific health profile. As demonstrated in Section 3.5.2, the integration of predictive models developed using data from fourteen different patients shows that the CIDSS has the flexibility to predict vital signs and, in turn, calculate the patient deterioration risk for various health profiles. This system has the ability to evolve and adapt to every patient condition since the first stage corresponds to using an ensemble of models to predict vital signs and the second stage corresponds to only using models with the lowest RMSE.

The early warning score calculation module uses vital sign records and determines the patient health deterioration based on a clinical protocol.

The CIDSS is also composed of three other modules: biometric sign error detection, basal value monitoring, and the communication manager.

The biometric sign error detection ensures the quality of all information concerning vital signs by validating, in a two-phase process, whether the vital sign values fall within the normal range for general COPD patients and subsequently, within the specific patient’s normal range using a probability density function.

The basal value monitoring analyzes the vital signs and suggests recommendations for new basal values to the patient if they deviate from the baseline provided by the HCAlert platform. The communication manager deals with all connections between the CIDSS modules, the HCAlert platform, and weather information sources.

The CIDSS system completed the white-box tests, including unit tests and integration tests.

All of these tests validate its functionality and contribution to preventing and potentially improving patient treatment by offering an early indication of the patient’s risk for deterioration.

Despite our ability to employ real-time telemonitoring patient data, we employed clinical historical longitudinal data that were gathered over a substantial period of time (2–3 years) through a telemonitoring application. This extended time frame enabled us to formulate conclusions regarding the system’s validity, supported by the early warning score implementation and the errors of the applied predictive models.

### 5.2. Limitations

The non-approval of the incorporation of new patients by the ethics committee associated with the HC PSI project made the testing and analysis of the CIDSS effectiveness in providing quality information regarding patient health deterioration risk difficult.

The scarcity of data was a limitation in our study, and two key aspects contributed to this challenge. Firstly, the measurements we had access to were not collected at hourly intervals, which restricted our ability to capture fine-grained variations in the data. The absence of hourly data points hindered our capacity to discern short-term patterns and trends, potentially hiding crucial insights that might have emerged with more frequent data collection.

Another significant data gap stemmed from the lack of information concerning home sensors, specifically data related to humidity levels. Humidity is a vital environmental factor that influences various aspects of indoor comfort, air quality, and overall well-being. The absence of the essential sensor data limited our ability to comprehensively assess the interplay between different environmental parameters, potentially leading to an incomplete understanding of the complex dynamics within the studied environment.

Despite the limitations, the system was validated, end-to-end, and clinically recognized as important for COPD monitoring, being adjustable enough to integrate these data sources if included in the project and handle a lower granularity of information to make predictions.

### 5.3. Future Work

As part of our future work, we will aim to identify some potential advancements to pursue. Firstly, we will aim to validate the effectiveness of the CIDSS (clinical deterioration surveillance system) by obtaining real-time patient data through the HCAlert platform. Analyzing these data over an extended period will help us assess the accuracy and quality of early information provided by the CIDSS, particularly regarding a patient’s risk of deterioration.

To enhance the robustness of our research, we will seek to access a more extensive and diverse dataset that includes patient data from different countries. Expanding our data collection to the international stage will ensure that our findings are relevant to a broader population.

Adopting a more inclusive approach involves considering a broader range of age-related values. By including individuals across various age groups, we could reveal some patterns and trends that may be present within different life stages.

To achieve more precise and detailed analyses, we propose incorporating more daily frequent recordings. This higher data capture frequency will enable us to detect subtle fluctuations and temporal dynamics that might be missed in less frequent sampling, providing real-time insights into patients’ vital signs.

Additionally, the integration of sensor technology to monitor indoor humidity levels would facilitate the extraction of valuable insights regarding the relationship between environmental factors and health deterioration.

By pursuing these advancements, we seek to increase the importance and reliability of our research, which could ultimately contribute to better patient treatment.

## Figures and Tables

**Table 1:** Topics of related work and their corresponding queries used to filter research papers related to each topic.

Subsection	Query
In-Home Healthcare for COPD	(“Healthcare Management Systems” AND “Real-time Detection”)
E-Healthcare supported by Predictive analytics	(“Healthcare Management Systems” AND “Early Detection” AND (“Artificial Intelligence” AND “Machine Learning”))
Factors related with COPD deterioration	(“Early Detection” AND “Vital Signs” AND “COPD”)
Machine Learning for for Early Identification of a Deterioration	(“Early Detection” AND “Vital Signs” AND “Machine Learning”)

**Table 2:** Eligibility criteria to filter research papers.

Eligibility Criteria	
**Inclusion Criteria**	**Exclusion Criteria**
Written in English or Portuguese	Not written in English or Portuguese
Publication date after/during 2010	Publication date before 2010

**Table 3:** Functional requirements associated with each module.

Requirements	Module
The predictive service should collect environmental data, such as air quality, seasonal infection incidences, and weather conditions	Vital Signs Prediction
The predictive service should correlate parameters and detect patterns	
The predictive service should reevaluate the weighting of each parameter, depending on the context (e.g., patient, clinical history, etc.)	
The collected data should undergo anonymization (if applicable), normalization, and data fusion	
The predictive service should consider the early warning score to generate alerts	Communication Manager
The predictive service should consider the alert classification to detect false positives	
The predictive service should advise the user to take a new measurement and launch inquiries to validate if it is a false positive	Biometric Sign Error Detection
The predictive system should apply the early warning score to the clinical protocol and suggest changes to the protocol based on the basal value	Early Warning Calculation
The predictive service should calculate the early warning score (define the correlation weighting of each parameter in the EWS calculation)	
The predictive system should recommend a reassessment of the basal value	Basal Value Monitoring
The predictive system should take into account changes made to the clinical protocol by the clinical team	
The predictive system should analyze the threshold for advising changes to the applied clinical protocol for the patient	

**Table 4:** Clinical information extracted from the Hope Care API.

idRawMeasurement	Measurement Identifier
createdOn	Measurement creation date
clientID	Identification of the patient to whom the measurement belongs
Latitude	Latitude of the patient
Longitude	Longitude of the patient
ProviderMNameStandard	Standard name of the type of measurement
Value	Measurement value
Unit	Units of measurement (in the dataset are available %, C, bpm, count, mmHg, NA, null, and percent)

**Table 5:** Weather historical information.

idWeatherMeasurement	Measurement Identifier
Station ID	Station identifier
Latitude	Latitude of the station
Longitude	Longitude of the station
Year	Year of the collected measurement
Month	Month of the collected measurement
Day	Day of the collected measurement
T_MED	Value of the daily mean temperature in Celsius
HR_MED	Value of the daily mean relative humidity in percent

**Table 6:** Historical information on air pollution.

idParticlesMeasurement	Measurement Identifier
Location	Location of the station
Latitude	Latitude of the station
Longitude	Longitude of the station
Date	Date of the collected measurement
PM10	Value of PM10
PM2_5	Value of PM2.5

**Table 7:** Root mean square error values for the 5th-day predictions of different model architectures trained using the dataset for the patient with ID no. 156.

Model	SpO2	Heart Rate	Systolic Blood Pressure	Body Temperature
ARIMA	2.080718	7.089329	9.783878	0.247163
XGBoost	0.817778	0.96435	2.407083	0.302518
LightGBM	0.064668	0.380769	2.170715	0.058705
GRU	0.083168	0.110159	0.130179	0.131379
LSTM	0.092241	0.573169	0.135822	0.137075
BILSTM	0.084948	0.113384	0.132097	0.130094

**Table 8:** Root mean square error values for the 5th-day predictions using the best model architectures trained on the dataset for the patient with ID no. 156.

Vital Sign Predicted	Type	RMSE
SpO2	LightGBM	0.064668
Heart Rate	GRU	0.110159
Systolic Blood Pressure	GRU	0.130179
Body Temperature	LightGBM	0.058705

**Table 9:** Early warning score clinical protocol suggested by Hope Care SA’s medical team.

	Description	0 Points	1 Point	2 Points	3 Points
SpO2	Difference between the predicted value for the day and the value from the previous day	<3%	3–5%	6–7%	>7%
Heart Rate	BPM Value	46–100	101–110	111–115	>115 or <46
Systolic Blood Pressure	Percentage difference between the predicted value for the day and the baseline value	<20%	≥20%	≥23%	≥25%
Body Temperature	Temperature value in Celsius	<37.5	37.5–37.9	38–38.4	>38.5

**Table 10:** Last 5 days of data extracted from the database for vital sign predictions on 25 April.

Date (yy-mm-dd)	Heart Rate (BPM)	Body Temperature (${}^{\circ }$C)	SpO2 (%)	Systolic Blood Pressure (mmHg)	T MED (${}^{\circ }$C)	HR MED (%)	PR QTD (mm)	pm25 (Count)
2022-04-20	60.0	37.1	96.0	92.0	9.68	51.30	0.11	0.82
2022-04-21	61.0	36.2	95.0	95.0	9.60	63.25	1.86	1.66
2022-04-22	63.0	36.0	95.0	93.0	7.53	82.97	23.25	0.93
2022-04-23	59.0	36.5	96.0	96.0	8.95	69.24	1.91	0.58
2022-04-24	65.0	36.2	96.0	100.0	10.79	67.82	0.29	1.14
2022-04-25	57.0	35.9	96.0	102.0	12.35	65.43	0.01	2.63

**Table 11:** Predicted vital sign values from 26 April to 30 April.

Date (yy-mm-dd)	SpO2 (%)	Heart Rate (BPM)	Systolic Blood Pressure (mmHg)	Body Temperature (Celsius)
2022-04-26	95.028053	63.863962	98.327346	36.244274
2022-04-27	94.801013	64.027884	98.783749	36.162657
2022-04-28	94.948091	64.413307	99.589877	36.218256
2022-04-29	95.127560	64.438053	99.516291	36.246443
2022-04-30	95.054558	64.429125	99.496265	36.196343

**Table 12:** Calculated values of the early warning score from 26 April to 30 April.

Date (yy-mm-dd)	SpO2 (%)	Heart Rate	Systolic Blood Pressure	Body Temperature
2022-04-26	0	1	0	0
2022-04-27	0	1	0	0
2022-04-28	0	1	0	0
2022-04-29	0	1	0	0
2022-04-30	0	1	0	0

**Table 13:** Root mean square error (RMSE) values of the top selected models for predicting the vital signs of patient 300.

Dataset Used to Train the Model	Model	Parameter	Value (RMSE)
304	BILSTM	Spo2	0.285014
181	GRU	Heart Rate	1.520008
184	BILSTM	Systolic Blood Pressure	1.904305
181	GRU	Body Temperature	0.250580

**Table 14:** Last 5 days of data extracted from the database for vital sign predictions on 25 October.

Date (yy-mm-dd)	Heart Rate (BPM)	Body Temperature (${}^{\circ }$C)	SpO2 (%)	Systolic Blood Pressure (mmHg)	T MED (${}^{\circ }$C)	HR MED (%)	PR QTD (mm)	pm25 (Count)
2022-10-20	68.0	35.40	96.0	96.0	14.47	82.26	10.73	1.42
2022-10-21	68.0	35.60	96.0	96.0	15.05	79.13	3.94	1.94
2022-10-22	74.0	35.80	96.0	96.0	14.91	74.00	18.72	1.20
2022-10-23	70.0	35.90	95.0	94.0	14.15	67.11	5.45	2.91
2022-10-24	72.0	35.80	97.0	93.0	14.32	72.58	1.47	1.93
2022-10-25	76.0	35.00	95.0	98.0	16.13	64.89	7.87	1.94

**Table 15:** Predicted vital sign values from October 26 October to 30 October.

Date (yy-mm-dd)	SpO2 (%)	Heart Rate (BPM)	Systolic Blood Pressure (mmHg)	Body Temperature (${}^{\circ }$C)
2022-10-26	96.386055	70.779388	95.078346	35.292265
2022-10-27	96.228622	72.117355	94.664948	35.597720
2022-10-28	96.208916	72.186485	94.973228	35.796912
2022-10-29	96.297836	73.253487	95.260201	35.886715
2022-10-30	96.020462	72.828354	96.042572	35.796912
2022-10-31	96.320145	71.559845	95.059273	35.292265

**Table 16:** Calculated early warning score values from 26 October to 30 October.

Date (yy-mm-dd)	SpO2 (%)	Heart Rate (BPM)	Systolic Blood Pressure (mmHg)	Body Temperature (${}^{\circ }$C)
2022-10-26	0	1	0	0
2022-10-27	0	1	0	0
2022-10-28	0	1	0	0
2022-10-29	0	1	0	0
2022-10-30	0	1	0	0
2022-10-31	0	1	0	0

**Table 17:** Results of the evaluation of the system by health professionals.

Criteria	Questions	Objective Statement	Eval 1	Eval 2
Clinical impact on patient treatment	Indicates the importance of an smart clinical decision support system capable of provide 5-day early warning scores for monitoring patients with COPD.	Importance of the intelligent clinical decision support system for monitoring patients with COPD.	5	5
Patients Life Quality Impact	Indicates the impact of a smart clinical decision support system, providing a 5-day early warning score on the quality of life of a patient with COPD.	Impact of a clinical intelligence decision support system on the quality of life of a patient with COPD.	5	5
Utility	Indicates the usefulness of a system for healthcare professionals; generates information whenever there are changes in patients’ baseline values.	Usefulness of a intelligent clinical decision support system that notifies about patient baseline value modifications.	4	5
	Indicates the importance of a system that provides short-time horizon (in minutes) early warning scores for the clinical follow-up of patients with COPD.	Importance of an intelligent clinical decision support system on the clinical follow-up of patients with COPD.	5	5
	Indicates the usefulness of a real-time alert system for healthcare professionals whenever an abnormal measurement occurs for a specific patient.	Usefulness of an intelligent clinical decision support system that notifies about abnormal measurement detections.	5	5
Consistency with the organization	Indicates the relevance of involving healthcare professionals in defining clinical intervals for abnormal measurements.	Clinical validation on the definition of intervals for abnormal measurements.	5	5
	Indicates the relevance of involving healthcare professionals in defining the formula for calculating the basal value.	Clinical validation on the definition of the basal value calculation formula.	4	5
	Indicates the relevance of involving healthcare professionals in selecting environmental and clinical parameters (e.g., vital signs) that most influence the clinical progression of patients with COPD.	Clinical validation on the selection of environmental and biometric signs that most influence the clinical progression of patients with COPD.	5	5
Integration with clinical protocols	Indicates the relevance of the adopted early warning score matrix for clinical decision-making and adjustment of therapeutic protocols for patients.	Relevance of the adoption of the early warning score matrix for the clinical decision-making and adjustment of therapeutic protocols for patients.	5	4

## Data Availability

Data are available upon request after approval from the Ethical Committee.
